# The effects of a plate model on the remission and need for hypoglycemic drugs of newly diagnosed type 2 diabetes in China: A randomized trial

**DOI:** 10.1016/j.pmedr.2023.102537

**Published:** 2023-12-06

**Authors:** Yongwen Zhang, Huanhuan Han, Lanfang Chu

**Affiliations:** a Department of Endocrinology, Nanjing Integrated Traditional Chinese and Western Medicine Hospital Affiliated with Nanjing University of Chinese Medicine, Nanjing 210014, China; b Department of Integrated Traditional Chinese and Western Medicine, General Hospital of Eastern Military Area, Nanjing 210012, China

**Keywords:** Type 2 diabetes, Plate model, Remission of diabetes, Hypoglycemic drugs, Low educational level, Prevention of diabetes

## Abstract

To assess the effect of the plate model on the remission of diabetes and the demand for hypoglycemic drugs in China. We selected 456 participants with newly diagnosed type 2 diabetes and not required to take hypoglycemic drugs at baseline. The plate education model consists of three parts: a colored leaflet suitable for low literacy reading, regular medical visits and health education sessions. The primary outcomes were remission of diabetes and the time to first use of hypoglycemic drugs. The study was ended after 8.1 years of follow-up. The incidence of the using hypoglycemic drugs was 36.15 % in the plate model, and 75.54 % in the low-fat model (*P* < 0.001). The prevalence of any remission in plate model was 27.1 % (95 % CI 16.8–37.4 %) during the first 2 years, decreasing to 14.5 % (95 % CI 6.3–22.7 %) during year 4, to 10.1 % (95 % CI 4.4–15.8 %) during year 6, and to 9.6 % (95 % CI 5.3–13.9 %) during year 8, compared with 12.2 % (95 % CI 5.2–19.2 %) at year 2, 6.1 % (95 % CI 2.1–10.1 %) at year 4, 4.7 %(95 % CI 2.2–7.2 %) at year 6, and 2.6 % (95 % CI 1.1–4.2 %) at year 8 in the low-fat group. The HbA1c of plate group was significantly decreased at the endpoint (7.74 ± 0.45 % vs. 6.70 ± 0.46 %, *P* < 0.001). The plate model may significantly improve the remission rate of diabetes, delay the demand for diabetes drugs, more suitable for patients with low educational level, and reduce the long-term level of HbA1c.

**Clinical trials registry:**

The study was registered at ChiCTR (www.chictr.org.cn) (ChiCTR1900027097).

## 1 Introduction

Several large randomized controlled studies on diabetes, including the Da Qing Diabetes Prevention Study (Da Qing study) (Li et al., 2008), the Finnish Diabetes Prevention Study (DPS) (Lindström et al., 2006), and the Diabetes Prevention Program (DPP) (Herman et al., 2005), demonstrate that medical nutrition therapy (MNT), a therapeutic lifestyle change characterized by individualized reduced-calorie meal plan, is highly effective in improving cardiometabolic markers and preventing type 2 diabetes (T2DM) (Esposito et al., 2010). Follow-up of three large trials of lifestyle intervention to prevent diabetes show that the risk of conversion to T2DM sustained reduction: the Da Qing study was 39 % reduction at 30 years (Li et al., 2008), the Finnish DPS was 43 % reduction at 7 years (Lindström et al., 2006), and the Diabetes Prevention Program Outcomes Study was 34 % reduction at 10 years (Perreault et al., 2019). The strongest evidence for diabetes prevention comes from the DPP study (Knowler et al., 2002); the DPP study shows that intensive lifestyle intervention can reduce the risk of developing T2DM by 58 % over 3 years. The intensive lifestyle intervention in DPP study was administered as a structured core curriculum, characterized by a more flexible maintenance program including group sessions, motivational campaigns, individual classes, and opportunities for restart.

For many patients with diabetes, the most challenging part of the diabetes management is determining what kind of diet pattern to choose. There is no “one size fits all” diet pattern for diabetes patients, and diet patterns should be individualized according sex, age, job scopes, educational levels and diet habits. MNT plays an indispensable role in overall management of diabetes, and every diabetes patient should actively participate in education, self-management, and development of treatment plans with their medical team, including the development of individualized diet patterns (Evert et al., 2014, Wing et al., 2013).

**Fig. 1:**
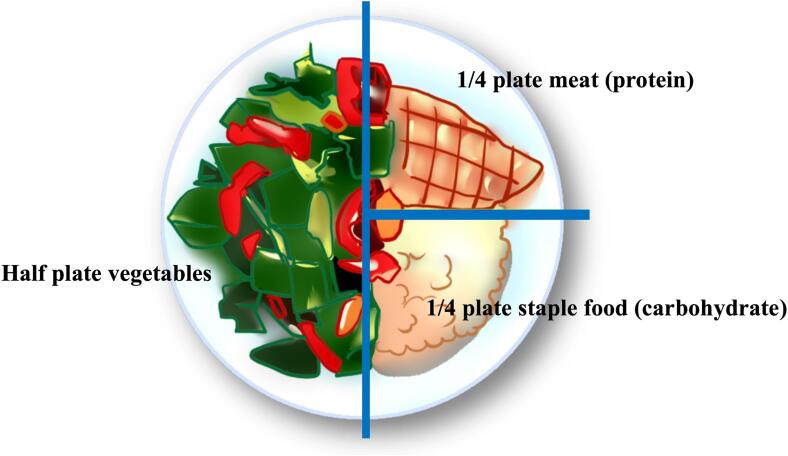
Schematic diagram of restricted diet with a plate used in Chinese adults with type 2 diabetes.

Given the complexity and difficulty of dietary and nutritional education for most diabetes patients, especially those with low educational levels, our team recommends a simplified approach called the “plate model” or “limited diet with plates” (Fig. 1) (Zhang and Chu, 2018, Zhang et al., 2022). A simplified education model has the potential to improve communication with those with lower educational levels by reducing reading needs (Marciano et al., 2019, Caruso et al., 2018). In our previous trials, the plate model is more effective than counting education, associated with less time for education, persists for a long time, promotes behavior change, allows dietary freedom, and less weight gain (Zhang and Chu, 2018, Zhang et al., 2022). In this trial, we assessed the effect of a plate model on the remission of diabetes and the demand for a first use of hypoglycemic drugs (either oral or injection) compared with a low-fat model with newly diagnosed patients with T2DM who were not required to take hypoglycemic drugs at enrollment.

## 2 Research design and methods

The study was designed as a randomized, multicenter, parallel-group trial. It was conducted in Nanjing aims to evaluate the effects of plate model versus low-fat model on the remission of diabetes and prevention of hypoglycemic drug therapy and cardiovascular risk markers. The study met the institution's guidelines for protection of human subjects concerning safety and privacy. The study was approved by the ethics committee of the Nanjing integrated traditional Chinese and western medicine hospital affiliated with Nanjing university of Chinese medicine. The study was conducted between October 2013 and December 2021, randomization was started in August 2013, and the trial ended after 8.1 years of follow-up. All study participants provided signed written informed consent. The study was registered at ChiCTR (https://www.chictr.org.cn) (ChiCTR1900027097).

### 2.1 Participants

Initially, we recruited 1,231 participants at enrollment, and excluded participants who received hypoglycemic drugs (n = 775). Finally, the current study only included data on participants with newly diagnosed T2DM and not required to take hypoglycemic drugs at baseline (n = 456). The inclusion criteria including: at least one counselling in our hospitals; age greater than 18 years and less than 65 years; do not require taking hypoglycemic drugs. The exclusion criteria including: HbA1c > 8.5 %, proteinuria (urinary albuminto-creatinine ratio ≥ 30 mg/mmol); renal insufficiency (eGFR < 60 mL/min); and involved in other trials. The computer-generated sequence of random number provided a random table for participants. Those participants were randomized at the baseline visit to a plate model group (n = 228) or a low-fat diet group (n = 228). The information in the food frequency questionnaire was used to estimate nutrient and energy intake. During the initial visit, each participant underwent a physical examination, including measurements of waist circumference, weight, blood pressure, and height. At the same time, the complications of diabetes were assessed, including myocardial infarction, hypertension, dyslipidemia, stroke, kidney disease and angina pectoris. Low-density lipoprotein cholesterol (LDL-C), total cholesterol (TC), triglycerides (TG), high-density lipoprotein cholesterol, HbA1c, fasting plasma glucose (FPG), 2-h postprandial glucose (2hPG), and uric acid were measured every six months after randomization.

### 2.2 Intervention

Randomization procedure was conducted by researchers independent of outcome evaluation and intervention implementation. The dietitians conducted group and individual dietary training at the baseline visit and quarterly thereafter for all participants. During each training session, a validated dietary questionnaire was used to evaluate participants’ adherence to different dietary patterns. The answers to questionnaires were used as an evaluation method to customize interventions for participants and to negotiate improvement measures to improve their dietary adherence. The plate model is a visual model to improve the adherence of diabetes through easy to understand pictures. We divide the plate into three parts, with a quarter of the plates containing fish, poultry, or meat; a quarter of the plates containing staple foods (carbohydrates), and the remaining half of the plates containing green vegetables (Zhang and Chu, 2018, Zhang et al., 2022). The plate education specifically designed for this study consists of three parts: a colored leaflet suitable for low literacy reading, which contains the composition and explanation of the plate model (Fig. 1); regular medical visits and health education sessions (Zhang et al., 2022). The dietary education for the low-fat diet group includes a paper booklet containing traditional low-fat diet education; regular health education sessions, and group medical visits. To promote the dietary compliance of the participants, the registered dietitians met with the patients individually every quarter and gave them individualized guidance. The dietitian provided professional suggestion and guidance on the dietary pattern, and reported patient's requirements and improvement measures to the study groups. We also recommended participants engage in at least 150 min of physical activity per week, but physical activity was not the focus of our study.

### 2.3 Outcomes measures

The primary outcomes were remission of diabetes and the time to first use of hypoglycemic drugs (oral or injectable). Secondary outcomes were cardiovascular risk markers, including glycemic control, body weight, lipids, and blood pressure. The researchers who collected information and assessed the outcomes were blind to the assumptions of the current study. If the HbA1c level of participants is >7%, an additional 3 months of adjustment time will be given to reinforce dietary control and increase physical activity; if the HbA1c level remained >7% after 3 months, participants reached the primary endpoint and the data will be censored.

Remission of diabetes was defined as FPG<7mmol/L, 2hPG<11.1 mmol/L, or HbA1c < 6.5 %. The partial remission of diabetes was defined as transition of blood glucose level from meeting diabetes criteria (FPG ≥ 7 mmol/L and (or) 2hPG ≥ 11.1 mmol/L, or HbA1c > 6.5 %) to prediabetes criteria (6.1 mmol/L ≤ FPG<7.0 mmol/L, 7.8 mmol/L ≤ 2hPG<11.1 mmol/L). Complete remission of diabetes was defined as the transition of blood glucose level from diabetes criteria to complete normalization (FPG<6.1 mmol/L, 2hPG<7.8 mmol/L).

### 2.4 Statistical analysis

All data analysis was conducted on the basis of intention to treat. Kaplan-Meier survival curves were described to estimate the probability of remaining free of diabetes medications, compared with two-sided log-rank test in the two groups. Using the low-fat group as a reference, calculated the hazard ratios (HRs) and their 95 % CIs. We conducted Cox regression analysis for time to introduction of hypoglycemic drugs, to evaluate the effectiveness of dietary interventions independent of weight loss. We compared the annual prevalence of any remission (partial or complete remission) between two groups and calculated the prevalence of sustained remission in years 2, 4, 6, and 8. We estimated the multivariable association of pre-determined demographic (educational level, age and sex) and baseline risk factors (diabetes duration, HbA1c, BMI, and physical activity) with any remission of diabetes. Laboratory data from baseline to multi-year of follow-up were modeled with generalized estimating equations and generalized linear regression. Person-years of follow-up were estimated from baseline to the earliest event (using hypoglycemic drugs), end of follow-up (December 30, 2021), or loss of follow-up. Participants lost during the follow-up process were considered as censored observations. In the subsequent multivariable model, we stratified according to educational levels, age, recruitment center and sex, and adjusted for diabetes duration, smoking status, alcohol intake, hypertension, dyslipidemia, waist-to-height ratio, MedDiet adherence, metabolic equivalent of task (METs), and moderate-to vigorous-intensity physical activity (MVPA). The Fisher exact test was used to analyze the prevalence of remission of diabetes and compare categorical safety variables. All statistical tests were two-sided, *P* < 0.05 was considered statistically significant. Statistical analyses were conducted using SPSS, version 25.0.

## 3 Results

We randomly divided 456 patients into the plate model and the low-fat model group. During the study period, the same number of patients withdrew from the trial. The baseline demographic and clinical characteristics of the two groups were similar, with approximately 50 % of patients had a low level of school education in both groups (Table 1).

**Table 1:** Baseline characteristics of Chinese adults with type 2 diabetes enrolled in the study in 2013.

**Variable**	**Plate model** **(n = 228)**	**Low-fat model** **(n = 228)**	***P* value**
Age(years), mean (SD)	47.10 (13.81)	48.25 (12.01)	0.780
Female sex, n (%)	116 (50.88)	109 (47.81)	0.512
Highest level of school education completed (%)			
Low level (less than junior high school)	107(46.92)	112(49.12)	0.639
Intermediate level (less than university degree)	71(31.14)	77(33.77)	0.548
High level (university degree or higher)	50(21.93)	39(17.11)	0.194
HbA1c, %	7.74 (0.45)	7.67 (0.47)	0.630
HbA1c, mmol/mol	55.52 (5.57)	53.55 (5.77)	0.630
FPG (mmol/l)	8.39(0.79)	8.31(0.73)	0.726
2hPG (mmol/l)	9.99(1.49)	10.55(1.54)	0.254
Uric acid (umol/L)	368.35(92.11)	372.10(73.11)	0.887
Diabetes duration (years)	2.51(1.38)	2.96(1.50)	0.325
F C-peptide (ng/mL)	1.92(1.04)	2.02(1.05)	0.775
HOMA2-IR	2.02(0.84)	2.11(0.65)	0.723
HOMA2-%B	71.80(16.65)	68.00(12.13)	0.414
Body weight (kg)	79.40(13.00)	76.85(14.62)	0.563
BMI (kg/m2)			
Mean (SD)	24.74 (3.76)	26.06(3.63)	0.263
<25, n (%)	89(39.04)	82(35.96)	0.498
25–30, n (%)	116(50.88)	112(49.12)	0.708
>30, n (%)	23(10.09)	34(14.91)	0.119
Smoking status, n (%)			
Never	87 (38.16)	80 (35.09)	0.496
Former	41(17.98)	46(20.18)	0.551
Current	100(43.86)	102(44.74)	0.850
Alcohol intake, n (%)			
Daily or almost daily	48(21.05)	55(24.12)	0.433
Special occasions only	84(36.84)	80(35.09)	0.696
Never	96(42.11)	93(40.79)	0.776
Waist circumference (cm)	83.82(6.07)	84.39(5.52)	0.760
Waist-to-height ratio	0.51(0.08)	0.50(0.08)	0.640
Diabetes Complications Severity Scale	1.59(0.54)	1.65(0.60)	0.741
Blood pressure, mmHg			
SBP	133.25(15.50)	131.50(19.41)	0.754
DBP	85.60(5.52)	83.05(8.44)	0.348
Lipids, mg/dL			
TC	249.85(69.32)	224.50(89.50)	0.323
LDL-C	167.00(72.15)	177.90(66.76)	0.623
HDL-C	55.45 (17.08)	59.10 (18.79)	0.524
TG	153.58(70.85)	140.58(76.91)	0.671
MedDiet adherence (14-point score)	7.20(1.91)	7.70(1.84)	0.404
MVPA (min/day)	44.45(23.82)	48.25(25.87)	0.632
METs	4.14(0.87)	3.91(1.13)	0.474
History of (%)			
Hypertension	97(42.54)	101(44.30)	0.705
Stroke	34(14.91)	30(13.16)	0.590
AMI	21(9.21)	26(11.40)	0.441
CHD Angina	49(21.50)	45(19.74)	0.643
Nephropathy	37(16.23)	41(17.98)	0.619
Antihypertension medications	99(43.42)	105(46.05)	0.572
Cholesterol-lowering medications	88(38.60)	81(35.52)	0.497

Data are mean ± SD or n (%);The waist-to-height ratio is waist circumference divided by height; MVPA, moderate-to vigorous-intensity physical activity; METs, metabolic equivalent of task; DBP, diastolic blood pressure; SBP, systolic blood pressure; CI, confidence interval; AMI, acute myocardial infarction; CHD, coronary heart disease.

### 3.1 Need for hypoglycemic drugs

**Fig. 2:**
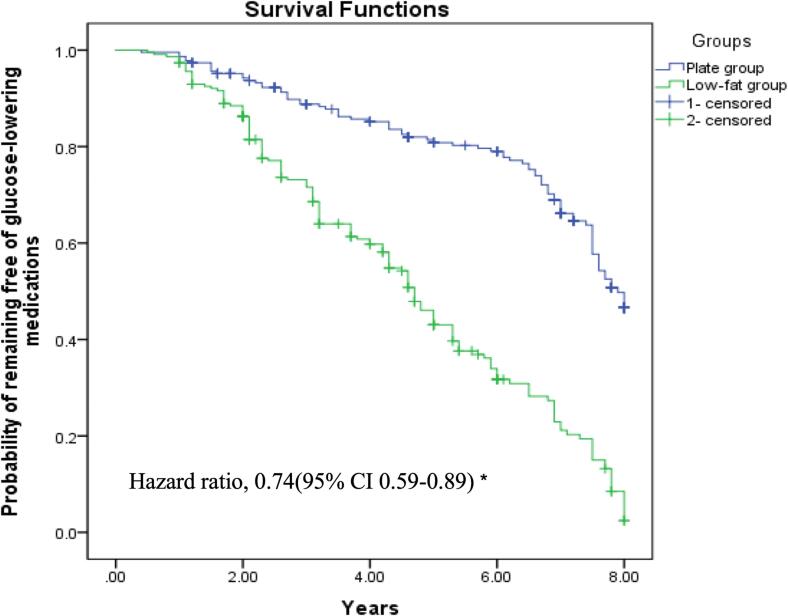
Kaplan-Meier estimate of the probability of remaining free of glucose-lowering medications in Chinese adults with type 2 diabetes during 8.1 years of follow-up in the study. *The Cox model was stratified according to sex, age, recruiting center, and educational level (three categories) and adjusted for hypertension (yes/no), diabetes duration (continuous), dyslipidemia (continuous), smoking status (never, former, or current), alcohol intake (daily or almost daily, special occasions only, never), waist-to-height ratio (continuous), MedDiet adherence(continuous), METs(continuous), and MVPA (continuous).

Fig. 2 shows the Kaplan-Meier survival curve of not taking hypoglycemic drugs in the two groups. After the end of our study, the cumulative incidence of the primary endpoint (using hypoglycemic drugs, either oral or injectable) was 36.15 % in the plate model, and 75.54 % in the low-fat model (*P* < 0.001). The corresponding unadjusted hazard ratio (HR) was 0.72 (95 % CI 0.58–0.86; *P* < 0.001), and the adjusted HR for risk factors was 0.74 (95 % CI 0.59–0.89; *P* < 0.001). The analysis with HbA1c > 7 % as the primary outcome yielded similar result (unadjusted HR 0.73 [95 % CI 0.58–0.88]; *P* < 0.001).

**Fig. 3:**
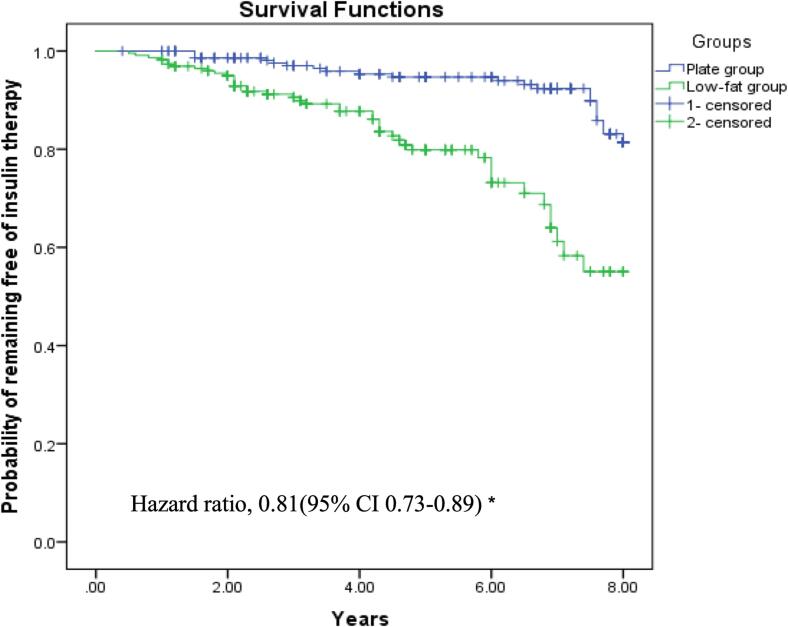
Kaplan-Meier estimate of the probability of remaining free of insulin therapy in Chinese adults with type 2 diabetes during 8.1 years of follow-up in the study. *The Cox model was stratified according to sex, age, recruiting center, and educational level (three categories) and adjusted for hypertension (yes/no), diabetes duration (continuous), dyslipidemia (continuous), smoking status (never, former, or current), alcohol intake (daily or almost daily, special occasions only, never), waist-to-height ratio (continuous), MedDiet adherence(continuous), METs (continuous), and MVPA (continuous).

Fig. 3 shows the Kaplan-Meier survival curve of remaining free of insulin treatment in the two groups. The unadjusted HR of free of insulin therapy was 0.80 (95 % CI 0.72–0.88) for the plate model compared with the low-fat model. The adjusted HR for the plate model was 0.81 (95 % CI 0.73–0.89) compared with the low-fat model. After 8.1 years of follow-up, the adjusted HR for starting to take hypoglycemic drugs was 0.84 (95 % CI 0.77–0.91) after an increase of 1-unit in the plate model questionnaire score.

### 3.2 Remission of diabetes

In all study years, the prevalence of complete remission was more common in the plate model than in the low-fat model. Plate model participants were significantly more likely to experience partial or complete remission, with a prevalence of 27.1 % (95 % CI 16.8–37.4 %) during the first 2 years, decreasing to 14.5 % (95 % CI 6.3–22.7 %) during year 4, to 10.1 % (95 % CI 4.4–15.8 %) during year 6, and to 9.6 % (95 % CI 5.3–13.9 %) during year 8, compared with 12.2 % (95 % CI 5.2–19.2 %) at year 2, 6.1 % (95 % CI 2.1–10.1 %) at year 4, 4.7 % (95 % CI 2.2–7.2 %) at year 6, and 2.6 % (95 % CI 1.1–4.2 %) at year 8 in the low-fat model. The remission ratios between the plate model and low-fat model varied from 2.8 (95 % CI 1.5–4.1) in year 2 to 2.6 (95 % CI 1.6–3.6) in year 4, 2.1 (95 % CI 1.4–2.8) in year 6, and 1.9 (95 % CI 1.1–2.7) in year 8.

### 3.3 Glycemic control

The changes in HbA1c are shown in Table 2. In the first two years of follow-up, participants in the plate model showed greater improvement in HbA1c levels compared to the low-fat model. The HbA1c of plate model was decreased by 0.66 % at the first two years, and significantly decreased at the endpoint (7.74 ± 0.45 % vs. 6.70 ± 0.46 %, *P* < 0.001). Participants in the low-fat model showed a 0.32 % decrease in HbA1c at the endpoint, but there was no statistically significant difference (7.67 ± 0.47vs. 7.36 ± 0.56 %, *P* = 0.061) (Table 2). In the plate model, FPG and 2hPG values significantly decreased at the endpoint (8.39 ± 0.79 % vs. 6.94 ± 0.82 %, *P* < 0.001; 9.99 ± 1.49 vs. 8.34 ± 1.39 %, *P* = 0.001) (Table 1 and Table 3).

**Table 2:** Mean values and changes of HbA_1c_ in Chinese adults with type 2 diabetes over 8.1 years of study.

**Time**	**Plate model (n = 228)** **(%, 95 % CI)**	**Low-fat model(n = 228)** **(%, 95 % CI)**	***P* value**
**HbA_1c_, unadjusted model**			
Baseline (n = 456)	7.74 (7.53 to 7.94)	7.67 (7.45 to 7.89)	0.630
2 years (n = 417)	7.09 (6.81 to 7.36)	7.59 (7.19to 7.99)	0.037
4 years (n = 342)	6.83 (6.55 to 7.11)	7.58 (7.23 to 7.89)	0.001
6 years (n = 286)	6.75 (6.50 to 6.99)	7.41 (7.14 to 7.67)	<0.001
8 years (n = 202)	6.70 (6.48 to 6.91)	7.36 (7.09 to 7.62)	<0.001
Baseline to 2 years	−0.66 (-0.92 to −0.39)	−0.08 (-0.48 to 0.32)	0.015
2 years to 4 years	−0.26 (-0.60 to 0.09)	−0.01 (-0.23 to 0.21)	0.553
4 years to 6 years	−0.09 (-0.30 to 0.13)	−0.17 (-0.37 to 0.35)	0.552
Baseline to 8 years	−1.05 (-1.37 to −0.72)	−0.32 (-0.61to −0.21)	0.001
**HbA_1c_, adjusted model**			
Baseline (n = 456)	7.74 (7.53 to 7.94)	7.67 (7.45 to 7.89)	0.630
2 years (n = 417)	7.09 (6.81 to 7.36)	7.59 (7.19to 7.99)	0.012
4 years (n = 342)	6.83 (6.55 to 7.11)	7.58 (7.23 to 7.89)	<0.001
6 years (n = 286)	6.75 (6.50 to 6.99)	7.41 (7.14 to 7.67)	<0.001
8 years (n = 202)	6.70 (6.48 to 6.91)	7.36 (7.09 to 7.62)	<0.001
Baseline to 2 years	−0.66 (-0.92 to −0.39)	−0.08 (-0.48 to 0.32)	0.010
2 years to 4 years	−0.26 (-0.60 to 0.09)	−0.01 (-0.23 to 0.21)	0.421
4 years to 6 years	−0.09 (-0.30 to 0.13)	−0.17 (-0.37 to 0.35)	0.435
Baseline to 8 years	−1.05 (-1.37 to −0.72)	−0.32 (-0.61to −0.21)	<0.001

The change of HbA1c was examined in longitudinal mixed models, based on an interaction between study group and time, and adjusted for age, sex, diabetes duration, HbA1c, FPG, 2hPG, educational level, hypertension, dyslipidemia, smoking status, alcohol intake, waist-to-height ratio, METs, blood pressure, MedDiet adherence, and MVPA.

**Table 3:** Changes of body weight, glycemic control, and cardiovascular risk markers in Chinese adults with type 2 diabetes during 8.1 years of follow-up in the study.

	**Plate model (n = 228)**		**Low-fat model (n = 228)**		*P* value*
	**Endpoint**	**Change**	**Endpoint**	**Change**	
Body weight (kg)	77.75 (12.57)	−1.65 (2.39)	75.65 (13.79)	−1.20 (2.35)	0.552
BMI (kg/m2)	23.95 (2.69)	−0.79 (1.54)	25.75 (3.52)	−0.25 (1.29)	0.241
Waist circumference (cm)	82.90 (6.13)	−0.92 (2.56)	83.90(5.29)	−0.50 (2.80)	0.478
FPG (mmol/L)	6.94(0.82)	−1.26 (1.11)	7.73 (0.60)	−0.58 (0.83)	0.034
2hPG (mmol/l)	8.34(1.39)	−1.72(1.05)	9.60(1.62)	−0.95(0.89)	0.016
CVD risk markers					
SBP (mmHg)	125.75 (14.94)	−7.00 (4.39)	127.95 (18.43)	−3.55 (4.86)	0.024
DBP (mmHg)	81.15 (9.28)	−4.35 (4.73)	80.25 (8.20)	−2.80 (5.59)	0.350
F C-peptide (ng/mL)	2.12 (0.97)	0.21 (0.32)	2.16 (0.99)	0.14(0.25)	0.482
HOMA2-IR	1.69 (0.59)	−0.33 (0.37)	1.81 (0.48)	−0.30 (0.29)	0.797
HOMA2-%B	76.25 (14.73)	4.85 (5.07)	72.60 (10.99)	4.60 (3.47)	0.857
TC (mg/dL)	237.20 (66.55)	−12.65 (11.25)	218.20 (85.74)	−6.35 (7.30)	0.042
LDL-C (mg/dL)	149.80 (56.69)	−12.20 (10.31)	172.95 (65.97)	−6.05 (5.30)	0.023
HDL-C (mg/dL)	62.55(19.88)	6.60(4.31)	68.15(18.51)	8.55(5.97)	0.244
TG (mg/dL)	146.50 (66.06)	−7.08 (11.27)	131.17 (71.86)	−7.75 (12.18)	0.891
MVPA (min/day)	51.70 (22.78)	7.25 (7.79)	56.90 (24.69)	10.15 (4.51)	0.158
METs	5.07(0.70)	0.94(0.47)	4.69(1.08)	0.73(0.56)	0.217
Education time (min/month)	17.80(2.62)		33.70(8.07)		<0.001

Data are means (SD), unless otherwise stated. * Changes between two groups.

### 3.4 Coronary risk factors and body weight

In the study, the weight changes of the two groups were-1.65 ± 2.39 and −1.20 ± 2.35, respectively. The absolute difference in weight loss was 0.45 kg, but there was no statistically significant difference between two groups (*P* = 0.552) (Table 3).

The plate model had a greater unadjusted effect on changes in LDL-C and TC levels, and there were statistically significant differences in the amount of increases between groups (−12.65 ± 11.25 % vs. −6.35 ± 7.30 %, *P* = 0.042; −12.20 ± 10.31 vs. −6.05 ± 5.30 %, *P* = 0.023). The low-fat model improved in all indicators, but there were no significant differences. BMI, waist circumference, HOMA2-IR, HOMA2-%B, MVPA, and METs were similar between the groups at the endpoint (*P* > 0.05) (Table 3).

### 3.5 Education time and blood pressure

There was a significant difference in educational time between the two groups of participants (17.80 ± 2.62 vs. 33.70 ± 8.07; *P* < 0.001). The baseline diastolic blood pressure (DBP) and systolic blood pressure (SBP) of the two models are similar (Table 1). The changes of SBP between two groups were statistically significant at the endpoint (−7.00 ± 4.39 vs. −3.55 ± 4.86; *P* = 0.024), DBP slightly decreased, and there was no statistically significant difference between the two groups. The low-fat model showed a decrease in DBP at the endpoint (−2.80 ± 5.59 mmHg), but there was no statistically significant difference (*P* > 0.05) (Table 3).

### 3.6 Physical activity

Changes in physical activity in groups are shown in Table 3. Both groups of participants increased their physical activity time (MVPA7.25 ± 7.79 vs 10.15 ± 4.51, *P* = 0.158; METs 0.94 ± 0.47 vs. 0.73 ± 0.56, *P* = 0.217), but there were no statistically significant differences in the amount of increase between the groups.

## 4 Conclusions

In the process of diabetes health education, we should pay more attention to people with low educational level. In our study, nearly half of the participants had a low level of education (less than junior high school), and a simplified dietary pattern makes it easier for these individuals with low educational level to understand and accept this pattern. However, plate model is not a single dietary pattern, it shares special features: popular and easy to understand, easy to persist for a long time, allows dietary freedom to most of individuals, and saves time for education. The results showed that compared to low-fat model, the plate model delayed the time of taking hypoglycemic drugs by 4.8 years, this impact was largely unrelated to weight loss and physical activity. In addition, 27.1 % of participants in the plate model had partial or complete remission of diabetes in the first two years after intervention, and 9.6 % of participants after 8 years, respectively, that is, from the blood glucose standard that met the diagnosis of diabetes to the prediabetes or normal blood glucose levels. These rates were two to three times higher than those of participants in the low-fat diet model. In the Look AHEAD trial (Wing et al., 2013), any remission rates were significantly higher among people who had a shorter duration of diabetes, substantial weight loss or increased physical activity, lower HbA1c levels and did not use insulin at recruitment. Since our study included different diabetes population, we were unable to evaluate the role of weight loss and diabetes duration, however, lower HbA1c level at entry, higher MedDiet adherence score, and more increase of MVPA and METs, which are important predictive factors for long-term remission.

Lifestyle intervention studies shows that a healthy lifestyle can postpone or delay the development of T2DM in patients with impaired grape regulation (from 30 % to 67 %) (Esposito et al., 2009, Salas-Salvadó et al., 2011, Esposito et al., 2003). In addition, a *meta*-analysis of 10 trials shows that participants with healthy dietary patterns had a 32 % reduction of risk of future T2DM (Esposito et al., 2010). Recently, a randomized trial on the prevention of cardiovascular events with a Med diet confirmed that compared to the control diet group, patients randomly received Med diet had an impressive magnitude of benefit (a reduction of approximately 30 % in cardiovascular disease) (Shai et al., 2008, Estruch et al., 2013).

In this trial, after a median follow-up of 5.2 years, compared with the low-fat diet group, the plate model significantly reduced the requirement of new-onset pharmacologic interventions. After a median follow-up of 3.1 years, the low-fat model group did not significantly reduce the rate of diabetes medications in newly diagnosed T2DM patients. The plate mode emphasizes a higher proportion of vegetables (recommended roots and green varieties), while the proportion of whole grains (grains, bread, rice or pasta) and red meat (recommended fish, chicken, and ducks) is relatively low (Zhang and Chu, 2018, Zhang et al., 2022). In addition to reducing the cardiovascular risk factors such as SBP, LDL-C and TC, our research results show that adopting plate model is more likely to maintain longer glycemic control, alleviate diabetes, and delayed hypoglycemic drugs demand (4.8 years). Newly diagnosed diabetes patients who do not take hypoglycemic drugs could significantly reduce medication costs, related adverse effects and hypoglycemia risk (Bolen et al., 2007, Budnitz et al., 2011). Even delaying the onset of diabetes will have a significant impact on the subsequent morbidity, thus have a substantial effect on the cost-effectiveness of diabetes prevention (Sofi et al., 2010, Murray and Lopez, 2013, Kendall et al., 2011).

The demand for hypoglycemic drugs (oral or injection) in the plate model group is relatively low, reflecting better glycemic control during the long-term follow-up period of the study, therefore, in order to maintain or achieve glycemic goals, the likelihood of using hypoglycemic drugs is lower. This beneficial effect may be due to the overall composition of the plate model leading to a decrease in calorie intake, long-term adherence to dietary control, and increased physical activity. After using robust variance estimators and adjusting for propensity scores, compared to the weight change of low-fat group (-1.20 kg, 95 % CI −0.42 to −1.98 kg), the average difference in weight change at 8.1 years in the plate group was −1.65 kg (95 % CI −0.65 to −2.55 kg). Although there was no statistically significant difference between the two groups, it is widely known that even mild weight loss can ultimately benefit patients in cardiovascular outcomes.

Our trial has certain limitations. Firstly, in our trial, the demand for hypoglycemic drugs was not a final endpoint. Therefore, these analyses are exploratory. Secondly, the patients participating in this study were inpatients or outpatient patients from our hospitals. Therefore, our findings may not be applicable to all diabetes patients with different dietary patterns in other regions. Thirdly, due to the inherent design of a dietary intervention trial using a holistic dietary pattern, this trial cannot be double-blind. Finally, due to the lack of a scale to study the impact of the plate model itself, we cannot determine the benefit levels of diabetes patients. Despite the above limitations, the advantages of our trial include adjustment of a series of potential confounding factors in multivariate analysis, long follow-up time, a wide range of participants in T2DM, and large sample size, which improves the reliability of our results.

In conclusion, our study results show that the rate of initiation of hypoglycemic drugs of participants in the plate model group is significantly lower than that in the low-fat group. This indicates that for newly diagnosed T2DM patients, the plate model may significantly reduce the long-term level of HbA1c, improve the remission rate of diabetes, more suitable for patients with low educational level, and delay the demand for hypoglycemic drugs.

## 5 Disclosure of ethical statements

• Approval of the research protocol: The protocol for the research project has been approved.

• Informed Consent: All study participants provided signed written informed consent.

• Approval date of Registry and the Registration No. of the study/trial: The study was registered at ChiCTR (https://www.chictr.org.cn) (ChiCTR1900027097).

• Animal Studies: N/A.

## CRediT authorship contribution statement

**Yongwen Zhang:** Formal analysis, Investigation, Methodology, Project administration. **Huanhuan Han:** Investigation. **Lanfang Chu:** Investigation.

## Declaration of competing interest

The authors declare that they have no known competing financial interests or personal relationships that could have appeared to influence the work reported in this paper.

## Data Availability

Data will be made available on request.
